# Development of an *In Vitro* Model of the Gut Microbiota Enriched in Mucus-Adhering Bacteria

**DOI:** 10.1128/spectrum.00336-23

**Published:** 2023-06-08

**Authors:** Marco Calvigioni, Adelaide Panattoni, Francesco Biagini, Leonardo Donati, Diletta Mazzantini, Mariacristina Massimino, Costanza Daddi, Francesco Celandroni, Giovanni Vozzi, Emilia Ghelardi

**Affiliations:** a Department of Translational Research and New Technologies in Medicine and Surgery, University of Pisa, Pisa, Italy; b Department of Information Engineering, University of Pisa, Pisa, Italy; c Research Center “Enrico Piaggio”, University of Pisa, Pisa, Italy; d Research Center “Nutraceuticals and Food for Health – Nutrafood”, University of Pisa, Pisa, Italy; University of Nebraska-Lincoln

**Keywords:** gut microbiota, gut model, mucins, mucus, adhesion

## Abstract

Culturing the gut microbiota in *in vitro* models that mimic the intestinal environment is increasingly becoming a promising alternative approach to study microbial dynamics and the effect of perturbations on the gut community. Since the mucus-associated microbial populations in the human intestine differ in composition and functions from their luminal counterpart, we attempted to reproduce *in vitro* the microbial consortia adhering to mucus using an already established three-dimensional model of the human gut microbiota. Electrospun gelatin structures supplemented or not with mucins were inoculated with fecal samples and compared for their ability to support microbial adhesion and growth over time, as well as to shape the composition of the colonizing communities. Both scaffolds allowed the establishment of long-term stable biofilms with comparable total bacterial loads and biodiversity. However, mucin-coated structures harbored microbial consortia especially enriched in *Akkermansia*, *Lactobacillus*, and *Faecalibacterium*, being therefore able to select for microorganisms commonly considered mucosa-associated *in vivo*.

**IMPORTANCE** These findings highlight the importance of mucins in shaping intestinal microbial communities, even those in artificial gut microbiota systems. We propose our *in vitro* model based on mucin-coated electrospun gelatin structures as a valid device for studies evaluating the effects of exogenous factors (nutrients, probiotics, infectious agents, and drugs) on mucus-adhering microbial communities.

## INTRODUCTION

As main components of the human mucus layer, mucins are highly glycosylated proteins produced by goblet cells. Due to their structure, which is widely rich in serine and threonine, they can link a huge number of oligosaccharides, resulting in very large three-dimensional (3D) glycoproteins (1). In the human intestine, mucins make contact with each other and with electrolytes, lipids, proteins, and other molecules secreted by intestinal cells to generate a thick viscoelastic mucus layer upon the epithelial cell stratum (2–4).

Under physiological conditions, mucus is almost impenetrable to microorganisms, which can only colonize its outer layer without the possibility of directly adhering to the underlying epithelial stratum (5). The ability to adhere to mucus is an advantageous property for microbes, protecting them from the flow-associated shear stress typical of the intestinal lumen and therefore allowing them to establish a stable colonization of the gut environment (5). It is well-documented that the mucus-associated intestinal microbial community is widely different in both richness and functions from its luminal counterpart due to deep gaps between the two ecological niches (6). Higher abundances of *Actinobacteria*, as well as *Clostridiales*, *Blautia*, and *Coprococcus*, were detected in the luminal microbiota (6), while more abundant colonization by *Firmicutes*, *Lachnospiraceae*, *Ruminococcaceae*, *Akkermansia*, *Bifidobacterium*, *Lactobacillus*, and *Faecalibacterium* was observed in the mucus-associated community (6–11).

While mucus can shape the composition of its adhered microbial community, conversely, the presence and biodiversity of the gut microbiota can influence the secretion, thickness, and maintenance of the mucus stratum (11). Among intestinal bacteria, a positive correlation with a healthy mucus has been shown for *Lactobacillus*, *Bifidobacterium*, *Allobaculum*, *Akkermansia*, *Faecalibaculum*, *Turicibacter*, and *Mucispirillum*, while *Proteobacteria* and some genera belonging to *Bacteroidetes* were shown to promote mucus permeability and impairment, consequentially triggering bowel inflammatory responses (12, 13). Given the presence of certain microbial species as a key factor in the overall mucus and well-being of the gut, detecting and identifying these beneficial bacteria have recently been pointed out as strategies to predict the potential risk of developing intestinal diseases and discomforts, especially those associated with mucus impairment and inflammation.

Considering the ethical restrictions in taking biological samples from humans and the frequent impossibility of translating results from animal models to humans, in the last decades, *in vitro* models faithfully mimicking the gut environment have become a promising alternative approach to obtaining information about the human gut microbiota (14). In complex artificial systems, environmental conditions (i.e., temperature, pH, oxygen, flow) can be controlled and biological components, such as mucus, can be added to recreate an environment more comparable to the physiological one (14). Moreover, the addition of mucus to *in vitro* models may promote adhesion and selection of physiologically mucus-associated bacteria (15, 16). Many studies on *in vitro* bacterial adhesion have evaluated the adhesive abilities of single microbial species in the presence of mucus (16–18). Nevertheless, how complex communities such as the gut microbiota adhere to mucins and how microbial compositional shifts are driven in the presence of mucus were scarcely tested.

In our previous works, we demonstrated the efficiency of a 3D *in vitro* model based on electrospun gelatin (EG) scaffolds in supporting the propagation and formation of biofilms by the gut microbiota, as well as in maintaining the biodiversity and richness of gut microbial communities (19, 20). Gelatin scaffolds showed better performance compared to traditional cultures, most likely because their three-dimensional reticular structure reproduces the complex pattern of bacterial interactions that characterizes the human gut and facilitates the survival of microbes after removal from the human host (19). In the present study, a mucin coating was added to these reticular scaffolds, increasing their spatial three-dimensional complexity and improving the *in vitro* model to better reproduce the mucosal environment found *in vivo* on the epithelial stratum. The ability of gut microbes to form biofilms on these scaffolds was assessed and the composition of the fecal microbiota grown *in vitro* in the presence of mucus investigated to verify whether selection for mucus-adhering bacteria occurred.

## RESULTS

### Biofilm formation on electrospun gelatin scaffolds.

**FIG 1:**
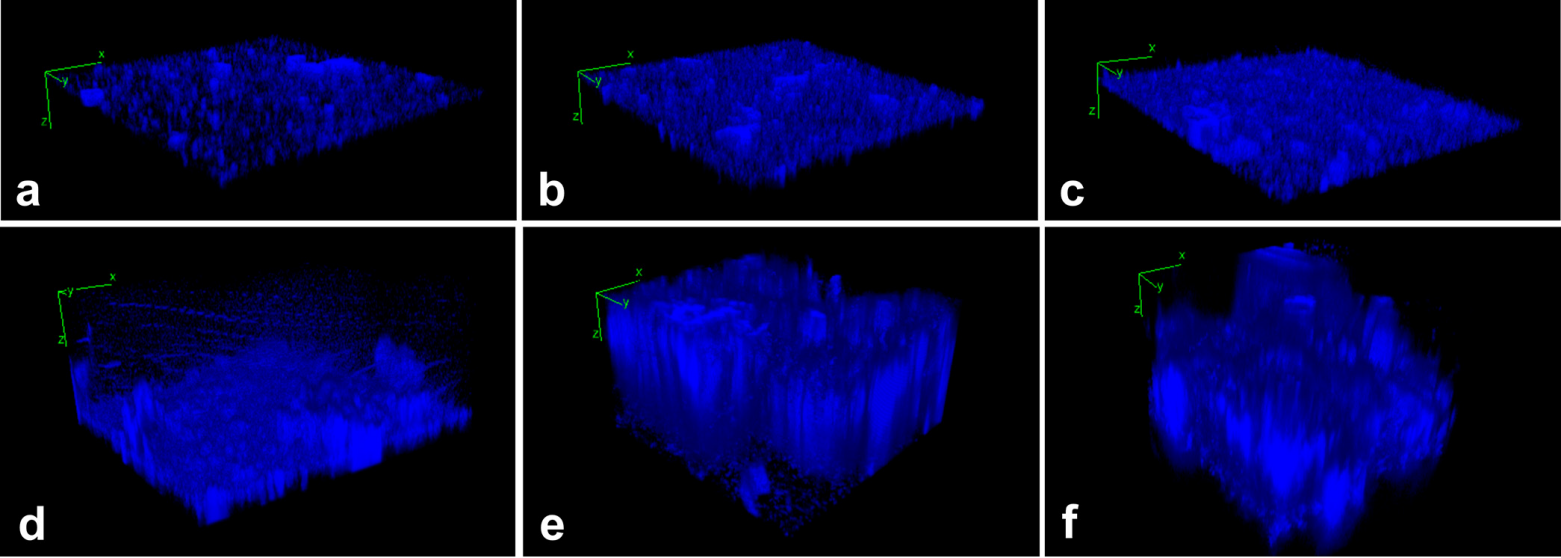
Confocal laser microscopy of biofilms formed on mucin uncoated (EG) and mucin-coated (EGM) electrospun gelatin structures. EG stained with DAPI at (a) 24 h, (b) 48 h, and (c) 72 h of incubation and EGM stained with DAPI at (d) 24 h, (e) 48 h, and (f) 72 h of incubation.

The ability of the fecal microbiota to form biofilms was tested on electrospun gelatin structures either coated (EGM) or not (EG) with mucins at different time points postinoculation. As already described in our previous work (20) and confirmed here using a slightly different protocol, the resulting gut microbiota were able to adhere to both mucin-coated and uncoated scaffolds. No statistically significant differences in adhered biomasses between EG and EGM were highlighted at each time point (optical density at 570 nm [OD_570_]: EG 24 h = 0.090 ± 0.006; EG 48 h = 0.127 ± 0.029; EG 72 h = 0.189 ± 0.016; EGM 24 h = 0.114 ± 0.023; EGM 48 h = 0.126 ± 0.043; EGM 72 h = 0.168 ± 0.013). Images obtained by confocal laser microscopy of DAPI (4′,6-diamidino-2-phenylindole)-stained biofilms grown on mucin-coated and uncoated membranes are shown in Fig. 1a to f.

### Absolute quantification of bacteria in the *in vitro* model.

**FIG 2:**
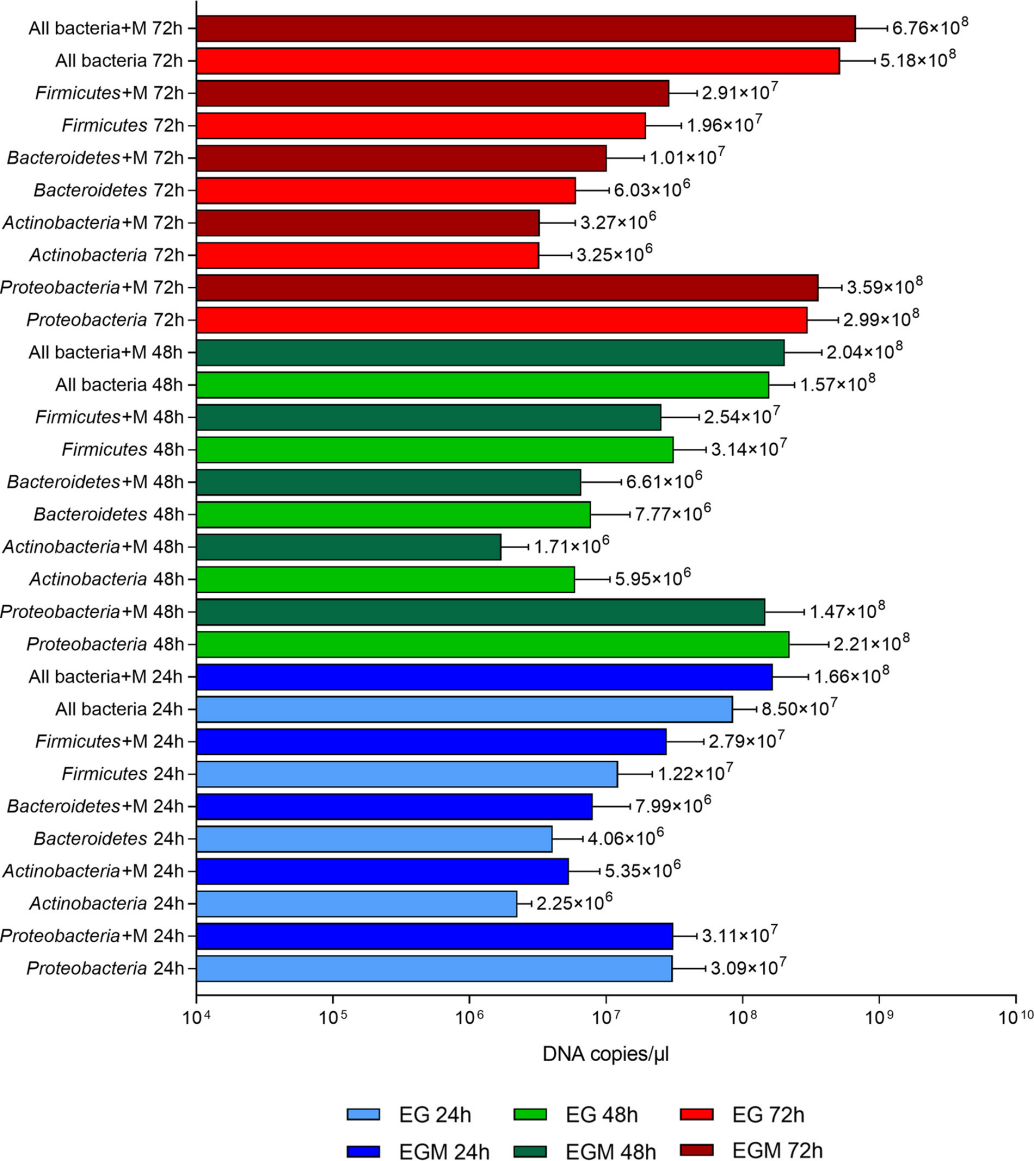
Analysis of microbial composition by real-time qPCR. Absolute abundances of the total bacterial load and main phyla (*Actinobacteria*, *Bacteroidetes*, *Firmicutes*, *Proteobacteria*) in fecal samples incubated for different times on electrospun gelatin structures in the presence (EGM 24 h, EGM 48 h, EGM 72 h; dark bars) and absence (EG 24 h, EG 48 h, EG 72 h; light bars) of mucins. The value on the right of each bar represents the mean value of the results obtained for each group.

Real-time quantitative PCRs (qPCRs) were performed to evaluate total bacterial load and the abundances of specific taxa following the *in vitro* growth of the fecal microbiota on EG and EGM scaffolds. The results obtained for EG scaffolds were comparable to those reported in a previous work (19) and no significant differences emerged in terms of total bacterial load and phyla composition between the two scaffolds (Fig. 2). The finding that the total amount of bacteria did not vary in the presence of mucins is consistent with the results obtained from biofilm quantification showing no differences between EG and EGM.

**FIG 3:**
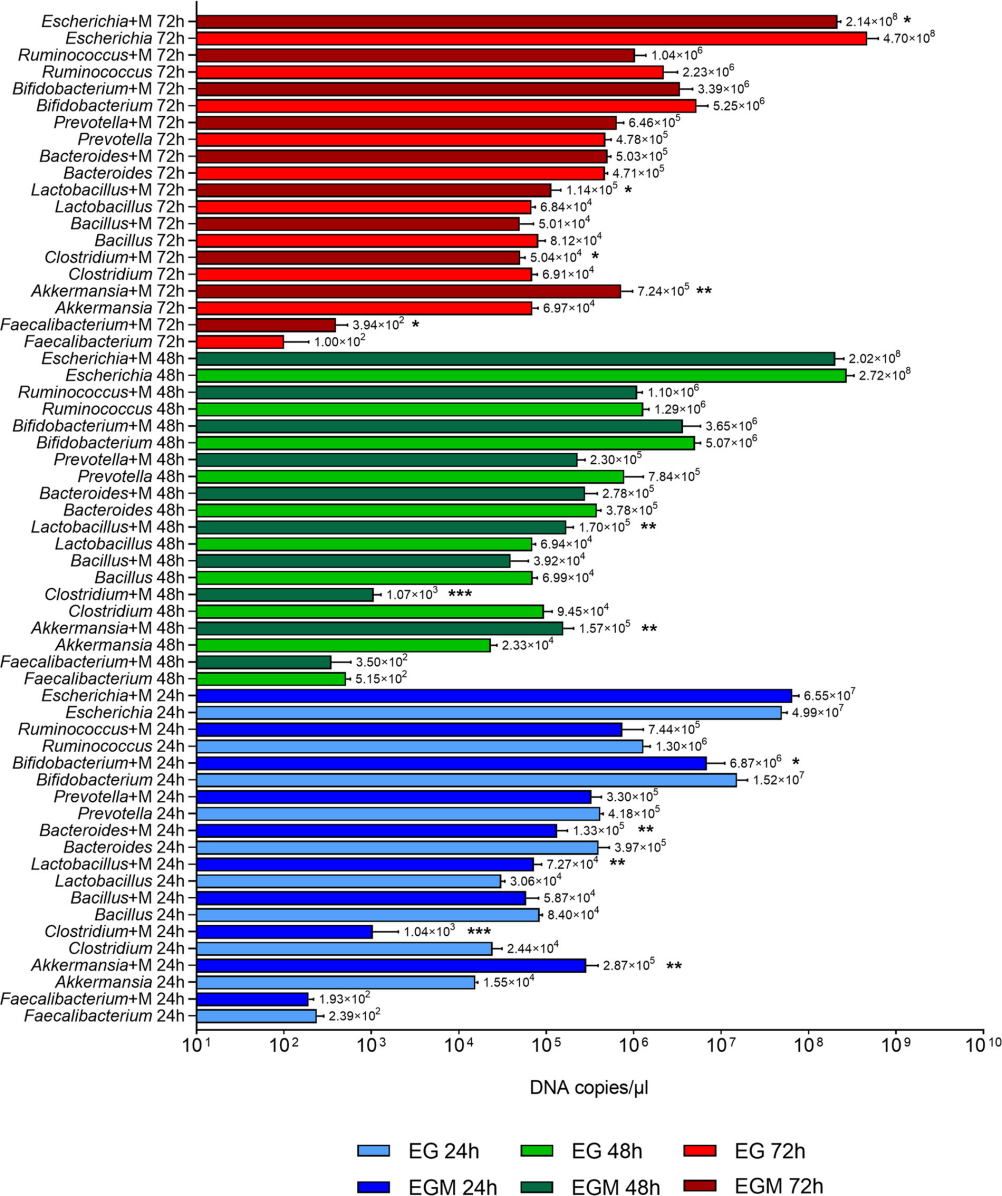
Analysis of microbial composition by real-time qPCR. Absolute abundances of *Akkermansia*, *Bacillus*, *Bacteroides*, *Bifidobacterium*, *Clostridium*, Escherichia, *Faecalibacterium*, *Lactobacillus*, *Prevotella*, and *Ruminococcus* in fecal samples incubated for different times on electrospun gelatin structures in the presence (EGM 24 h, EGM 48 h, EGM 72 h, dark bars) and absence (EG 24 h, EG 48 h, EG 72 h, light bars) of mucins. The value on the right of each bar represents the mean value of the results obtained for each group. *, *P* < 0.05; **, *P* < 0.01; ***, *P* < 0.001.

Although no differences were highlighted at the phylum level, interesting variations in the absolute abundances of bacterial genera were observed between EG and EGM (Fig. 3). *Akkermansia* (24 h, *P* = 0.0021; 48 h, *P* = 0.0015; 72 h, *P* = 0.0020) and *Lactobacillus* (24 h, *P* = 0.0020; 48 h, *P* = 0.0011; 72 h, *P* = 0. 0325) showed remarkable higher abundances on EGM structures at each time point. Conversely, the abundance of *Clostridium* was always lower on EGM than on EG (24 h, *P* = 0.0006; 48 h, *P* = 0.0002; 72 h, *P* = 0.0209). *Bifidobacterium* (*P* = 0.0400) and *Bacteroides* (*P* = 0.0088) decreased on the mucin-coated scaffolds after 24 h of incubation, while they showed no differences in their abundances at 48 h and 72 h. An increase in *Faecalibacterium* was detected at 72 h on EGM (*P* = 0.0134), while Escherichia*-Shigella* showed the opposite behavior, being less abundant at the same time point (*P* = 0.0171). In contrast, no mucin-dependent variations emerged for *Bacillus*, *Prevotella*, or *Ruminococcus*.

The overall quantitative results obtained using EGM highlight the efficacy of mucin-coated gelatin scaffolds in enriching the fecal microbiota in microorganisms commonly considered mucus-adhering, such as those belonging to the genera *Akkermansia*, *Lactobacillus*, and *Faecalibacterium*.

### Evaluation of bacterial biodiversity and richness in the *in vitro* model.

**FIG 4:**
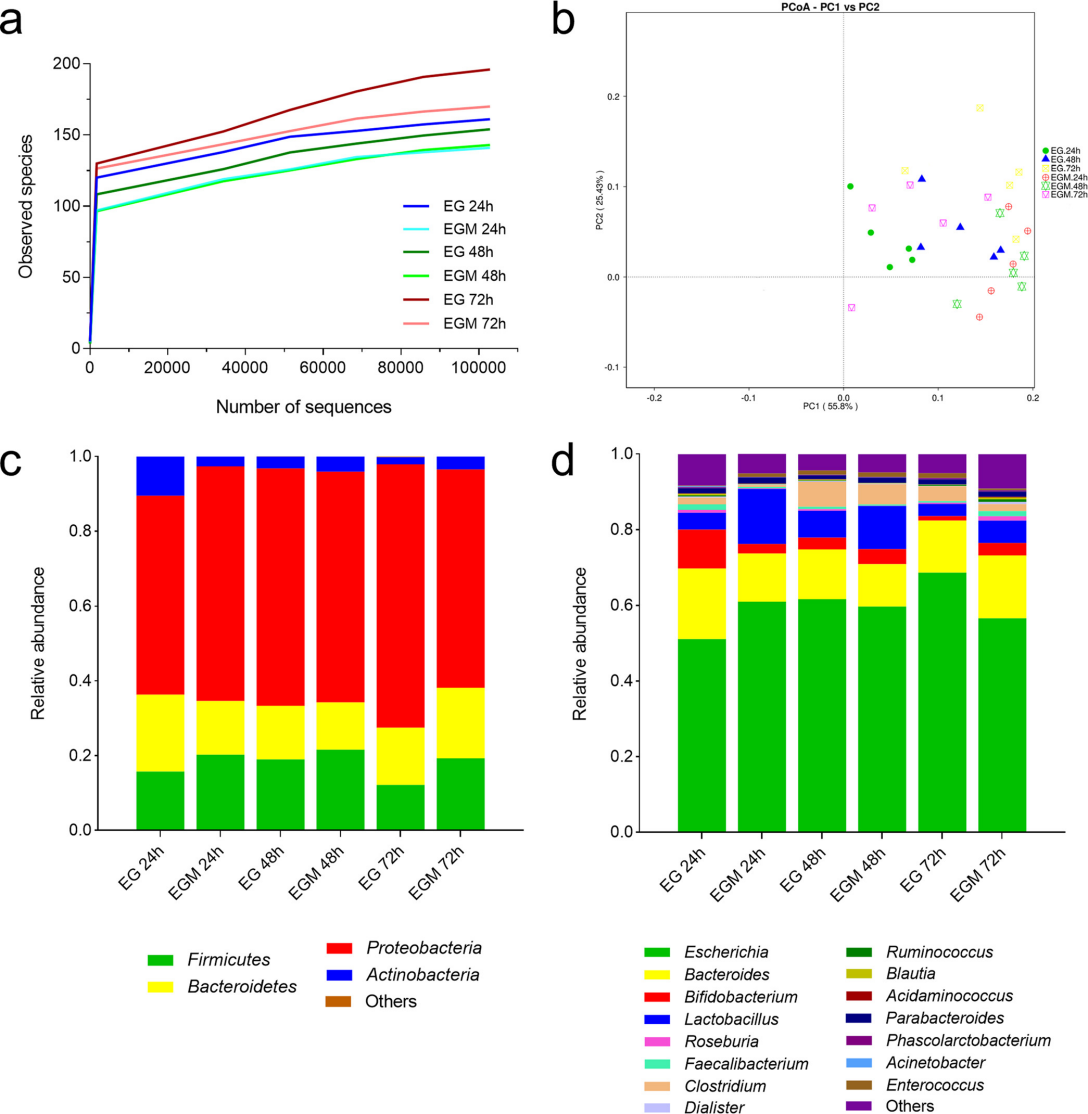
16S rRNA gene-based metagenomic analysis from fecal samples incubated for different times on electrospun gelatin structures in the presence (EGM 24 h, EGM 48 h, EGM 72 h) and absence (EG 24 h, EG 48 h, EG 72 h) of mucins. (a) Rarefaction curves. (b) PCoA plot. (c) Relative abundances of phyla. (d) Relative abundances of genera.

DNA extracted from EG and EGM scaffolds was subjected to 16S rRNA gene sequencing and metagenomic analysis to qualitatively evaluate the overall bacterial distribution on mucin-coated scaffolds and scaffolds alone. Rarefaction curves revealed comparable operational taxonomic unit (OTU) richness among all groups, with a maximum of 196 identified OTUs obtained from the microbial consortia grown on EG structures at 72 h (Fig. 4a). In terms of beta-diversity, principal-coordinate analyses (PCoA) did not reveal any significant clustering of samples (Fig. 4b).

Data obtained from metagenomic analyses concerning the microbial composition were compliant with the absolute abundances obtained by real-time qPCR (Fig. 3), showing an increase of *Lactobacillus* at all time points and *Faecalibacterium* at 72 h, as well as a reduction of *Clostridium* at all time points, *Bifidobacterium* and *Bacteroides* at 24 h, and Escherichia at 72 h on mucins (Fig. 4c and d).

To summarize the composition of the *in vitro*-grown microbiota, Table 1 shows the 20 most abundant genera and species found on EG and EGM at 24, 48, and 72 h. Some genera and species were detected in all groups (Table 1, marked in bold), while others only in some groups (Table 1). Despite the *Clostridium* genus being less abundant on EGM, C. perfringens was among the 20 most abundant species on these scaffolds at 48 and 72 h of incubation.

**TABLE 1:** List of the 20 most relevant bacterial genera and species found in fecal samples incubated for different times on electrospun gelatin structures in the presence and absence of mucins^*a*^

Sample duration (h) and type	Most abundant genera	Most abundant species
24 h		
EG	Acinetobacter, *Alistipes*, ***Bacteroides***, ***Bifidobacterium***, ***Blautia***, **unidentified *Burkholderiaceae***, *Catenisphaera*, ***Clostridium***, *Dialister*, *Dorea*, **Escherichia**, ***Faecalibacterium***, unidentified *Lachnospiraceae*, ***Lactobacillus***, *Mitsuokella*, ***Parabacteroides***, *Roseburia*, *Ruminococcus*, ***Subdoligranulum***, ***Sutterella***	Acinetobacter johnsonii, Bacteroides caccae, **Bacteroides cellulosilyticus**, **Bacteroides dorei**, **Bacteroides massiliensis, Bacteroides ovatus**, **Bacteroides thetaiotaomicron**, **Bacteroides uniformis**, **Bifidobacterium adolescentis**, **Clostridium butyricum**, Coprococcus comes, Dorea longicatena, **Escherichia coli**, **Lactobacillus ruminis**, **Parabacteroides distasonis**, **Parabacteroidesmerdae**, **Phascolarctobacterium faecium**, Roseburia faecis, Roseburia inulinivorans, **Sutterella wadsworthensis**
EGM	*Allisonella*, *Alistipes*, ***Bacteroides***, ***Bifidobacterium***, ***Blautia***, **unidentified *Burkholderiaceae***, ***Clostridium***, *Dialister*, *Dorea*, *Enterococcus*, **Escherichia**, ***Faecalibacterium***, ***Lactobacillus***, *Mitsuokella*, ***Parabacteroides***, *Phascolarctobacterium*, Pseudomonas, *Roseburia*, ***Subdoligranulum***, ***Sutterella***	*B. caccae*, **B. cellulosilyticus**, ***B. dorei***, ***B***, ***massiliensis***, ***B. ovatus***, **B. thetaiotaomicron**, **B. uniformis**, **B. adolescentis**, ***C. butyricum***, *C. comes*, *D. longicatena*, Enterococcus faecalis, **E. coli**, ***L. ruminis***, **P. distasonis**, ***P. merdae***, ***P. faecium***, Pseudomonas aeruginosa, *R. faecis*, ***S. wadsworthensis***
48 h		
EG	*Allisonella*, ***Bacteroides***, ***Bifidobacterium***, ***Blautia***, **unidentified *Burkholderiaceae***, ***Clostridium***, *Dialister*, *Dorea*, *Enterococcus*, **Escherichia**, ***Faecalibacterium***, unidentified *Lachnospiraceae*, ***Lactobacillus***, *Mitsuokella*, ***Parabacteroides***, Pseudomonas, *Roseburia*, *Ruminococcus*, ***Subdoligranulum***, ***Sutterella***	*B. caccae*, **B. cellulosilyticus**, ***B. dorei***, ***B. massiliensis***, ***B. ovatus***, **B. thetaiotaomicron**, **B. uniformis**, **B. adolescentis**, ***C. butyricum***, *C. comes*, *D. longicatena*, E. faecalis, **E. coli**, ***L. ruminis***, **P. distasonis**, ***P. merdae***, ***P. faecium***, P. aeruginosa, *R. faecis*, ***S. wadsworthensis***
EGM	Acinetobacter, *Allisonella*, ***Bacteroides***, ***Bifidobacterium***, ***Blautia***, **unidentified *Burkholderiaceae***, ***Clostridium***, *Dialister*, *Enterococcus*, **Escherichia**, ***Faecalibacterium***, ***Lactobacillus***, *Mitsuokella*, ***Parabacteroides***, *Phascolarctobacterium*, Pseudomonas, *Ruminococcus*, ***Subdoligranulum***, ***Sutterella***, *Veillonella*	*A. johnsonii*, *B. caccae*, **B. cellulosilyticus**, ***B. dorei***, ***B. massiliensis***, ***B. ovatus***, **B. thetaiotaomicron**, **B. uniformis**, **B. adolescentis**, ***C. butyricum***, Clostridium perfringens, E. faecalis, **E. coli**, ***L. ruminis***, **P. distasonis**, ***P. merdae***, ***P. faecium***, P. aeruginosa, Ruminococcus bromii, ***S. wadsworthensis***
72 h		
EG	*Allisonella*, ***Bacteroides***, ***Bifidobacterium***, ***Blautia***, **unidentified *Burkholderiaceae***, ***Clostridium***, *Dorea*, *Enterococcus*, **Escherichia**, ***Faecalibacterium***, *Lachnoclostridium*, unidentified *Lachnospiraceae*, ***Lactobacillus***, ***Parabacteroides***, *Phascolarctobacterium*, Pseudomonas, *Roseburia*, *Ruminococcus*, ***Subdoligranulum***, ***Sutterella***	**B. cellulosilyticus**, ***B. dorei***, ***B. massiliensis***, ***B. ovatus***, **B. thetaiotaomicron**, **B. uniformis**, **B. adolescentis**, ***C. butyricum***, *C. comes*, *D. longicatena*, E. faecalis, **E. coli**, ***L. ruminis***, **P. distasonis**, ***P. merdae***, ***P. faecium***, P. aeruginosa, *R. faecis*, *R. bromii*, ***S. wadsworthensis***
EGM	***Bacteroides***, ***Bifidobacterium***, ***Blautia***, **unidentified *Burkholderiaceae***, ***Clostridium***, *Dialister*, *Dorea*, *Enterococcus*, **Escherichia**, ***Faecalibacterium***, *Lachnoclostridium*, *Lachnospira*, unidentified *Lachnospiraceae*, ***Lactobacillus***, *Mitsuokella*, ***Parabacteroides***, *Roseburia*, *Ruminococcus*, ***Subdoligranulum***, ***Sutterella***	**B. cellulosilyticus**, ***B. dorei***, ***B. massiliensis***, ***B. ovatus***, **B. thetaiotaomicron**, **B. uniformis**, **B. adolescentis**, ***C. butyricum***, C. perfringens, *C. comes*, *D. longicatena*, E. faecalis, **E. coli**, ***L. ruminis***, **P. distasonis**, ***P. merdae***, ***P. faecium***, *R. faecis*, *R. bromii*, ***S. wadsworthensis***

a EG, electrospun gelatin structures; EGM, EG coated with mucin. Bold text indicates genera and species detected in all groups.

Less abundant but relevant intestinal genera (i.e., *Acidaminococcus* and *Eubacterium*) and species (i.e., Acidaminococcus intestini, Alistipes indistinctus, Alistipes obesi, Alistipes onderdonkii, Alistipes shahii, Bacteroides clarus, Bacteroides eggerthii, Bacteroides fragilis, Bacteroides nordii, Bacteroides stercoris, Bifidobacterium animalis, Bifidobacterium bifidum, Clostridium clostridioforme, Clostridium disporicum, Clostridium scindens, Clostridium symbiosum, Eubacterium hallii, Eubacterium ramulus, Eubacterium ventriosum, Lactobacillus casei, Parabacteroides goldsteinii, Ruminococcus bicirculans) were found, confirming the ability of the used *in vitro* model to ensure the growth and persistence of important microbes inhabiting the human intestine.

## DISCUSSION

The gut microbiota can establish biofilms on the intestinal mucosa, especially on the surface of the mucus layer and within its outer-half thickness (21), because mucins can provide an ideal substrate for adhesion and growth of certain microbial species (22). Previous studies have indicated that microbial consortia residing in the gut can also constitute biofilms *in vitro* (23, 24). In addition, the gut microbiota can form 3D multilayered biofilms on electrospun gelatin scaffolds, as shown by confocal laser and electric scanning microscopy (19, 20). In this work, we demonstrate the ability of the gut microbiota to form biofilms on mucin-coated electrospun gelatin structures and that the total amount of adhered microorganisms is maintained over time and does not differ from that found on mucin-free scaffolds. Therefore, mucins appear not to affect the overall adhesive ability of the microbial community, but rather to be able to shape its composition in terms of genera and species.

Mucus has a role in driving shifts in the composition of gut microbial populations *in vivo*, creating niches where different microbial clusters can establish (25). In line with these observations, our results show that mucins can induce changes in the fecal microbiota toward an increase in mucus-associated bacteria (i.e., *Akkermansia*, *Lactobacillus*, and *Faecalibacterium*) also *in vitro*. Akkermansia muciniphila is well-known to bind mucins and degrade them as a carbon, nitrogen, and energy source (26), as demonstrated by the presence of several genes encoding mucolytic enzymes in its genome (27). This behavior was also observed *in vitro*, with A. muciniphila increasing when mucins were added to the culture medium (28, 29). The tendency of *Lactobacillus* spp. to grow better on mucus has already been described (15, 29, 30). Lactobacilli have been reported to produce several mucin-targeting adhesion factors, including pili, mucus-binding proteins, and moonlighting proteins (31, 32). The few available studies on the adhesion of *Faecalibacterium* to mucins are not unanimous about its adhesive behavior. The ability of F. prausnitzii CNCM I-4546 and CNCM I-4573 to adhere to mucins in anaerobic conditions was confirmed (33), while *F. prausnitzii* ATCC 27766 displayed higher adhesive properties in the absence of mucus (34). Nevertheless, it is well-known that the mucosal environment is enriched in *Faecalibacterium* spp. (11, 35)., which play positive roles in maintaining a healthy mucosa-associated microbiota (35) and in regulating mucin production and glycosylation (36). The observation that the amount of *Faecalibacterium* only increased after 72 h of incubation on EGM scaffolds could be explained by its direct adhesion to mucins or the establishment of cross-feeding interactions with other microbial species able to sustain its expansion in later times. In fact, because some species residing in the gut demonstrate evident mucolytic properties (e.g., *A. muciniphila*, *Bacteroides* spp., *Enterococcus* spp., Ruminococcus gnavus), mucus-derived metabolites can be exploited by other microbes, including *Faecalibacterium* spp., for their own growth and persistence (37–39). The finding that the mucus-adhering microorganisms *Akkermansia* and *Lactobacillus* were able to colonize the mucin-coated structures within the first hours of incubation could also suggest their activity as pioneers in the modification of the mucus environment, thus allowing other microbes (i.e., *Faecalibacterium*) to settle and form an adapted community.

In this study, although a total reduction in *Clostridium* spp. was observed, suggesting a poor tendency of this genus to adhere to mucins, *C*. *butyricum* and C. perfringens were among the 20 most abundant species on mucin-coated scaffolds. This result can be explained by the recent demonstration that *C. butyricum* can adhere to mucins, modulate their glycosylation profile, and induce mucus secretion from HT-29 cells (40). In addition, the ability to encode a variety of carbohydrate-degrading enzymes able to hydrolyze the glycans constituting the mucus layer has been demonstrated for C. perfringens (41, 42), despite the fact that no evidence regarding its adhesive properties on mucins has been found.

In conclusion, the standardized *in vitro* model described in this study appears able to highlight the adhesive ability to mucins of different species residing in the gut. While no differences resulted from mucin addition in terms of biofilm formation, the *in vitro*-grown microbiota displayed different microbial compositions when mucus was added. The described culture system was effectively able to maintain the richness and biodiversity of the cultured microbial populations, despite shaping their composition and selecting for microbial genera and species commonly associated with the mucosal environment, such as *Akkermansia*, *Lactobacillus*, and *Faecalibacterium.* However, since stool samples collected from different individuals probably contain different microbial populations in terms of quality and the amount of inhabiting microorganisms, the adhesive behaviors of bacteria to the electrospun gelatin structures could substantially differ from those observed with the samples from our donor. Using fecal samples from different individuals, this model could be useful to recreate the microbial communities residing *in vivo* within the mucus layer, without the need for invasive intestinal biopsies. Nutrients, probiotics, infectious agents, and drugs could also be added to the mucin-supplemented model to evaluate their effects on the mucus-associated microbial communities. In addition, this *in vitro* model could help in the comprehension of the microbiota inhabiting the intestinal mucosa in healthy and diseased individuals, thus opening new perspectives for targeted preventive and therapeutic strategies to manage diseases, especially those associated with mucus impairment and intestinal inflammation.

## MATERIALS AND METHODS

### Preparation of raw and mucin-coated electrospun gelatin scaffolds.

The biofabrication protocol of the electrospun gelatin structures is described in detail in our previous work (19). EG sheets were first cut into circles with a diameter of 15 mm. Round scaffolds were inserted in sterile flat-bottomed 24-well microplates (Thermo Fisher Scientific, USA) and sterilized using 70% (vol/vol) ethanol (Thermo Fisher Scientific) for 15 min in a sterile environment. After ethanol was removed, structures were exposed to UV light for an additional 15 min in a sterile environment and air-dried. Next, 200 μL of a previously autoclaved suspension made of 5% (wt/vol) mucins (type II mucins from porcine stomach, containing MUC2; Merck KGaA, Germany) in sterile water was added and the mixture was incubated overnight at 4°C to guarantee proper immobilization of mucins upon EG structures (43). After incubation, wells were washed three times with 1 mL sterile phosphate-buffered saline (PBS; 5 M NaCl, 1 M KH_2_PO_4_, 1 M K_2_HPO_4_ [pH 7.2]) to remove unbound proteins, resulting in electrospun gelatin mucin-coated scaffolds.

### Microbial growth on the scaffolds.

A voluntary fecal sample donor was selected as previously reported (19) and stools were prepared following the European Guidelines for fecal microbiota transplantation (44). Aliquots of fecal suspensions were stocked at −80°C in 10% vol/vol glycerol until use. Next, 100 μL of fecal suspensions was inoculated on the sterile EG and EGM structures in the 24-well microplates. RPMI 1640 medium (Merck KGaA) was added to a final volume of 2 mL per well. Sterile control wells, consisting of sterile EG or EGM structures and RPMI 1640 in the absence of the fecal microbiota, were also included. Separate plates were incubated for different time points (i.e., 24, 48, and 72 h postinoculation) at 37°C in an anaerobic atmosphere generated using AnaeroGen Compact (Thermo Fisher Scientific). For plates incubated for 48 and 72 h, 670 μL of supernatant was replaced daily with an equal volume of fresh medium.

### Biofilm biomass measurement.

Adhered biomasses on EG and EGM structures were quantified by a crystal violet assay. Microbial suspensions were removed, and each well was washed three times with 1 mL PBS to ensure the removal of non-adhered microorganisms. Next, 2 mL of 0.1% (wt/vol) crystal violet (Carlo Erba, Italy) was added to stain the biofilms. Wells were incubated for 30 min at room temperature and washed three times with 1 mL deionized water. Two mL of absolute ethanol was subsequently added to solubilize the crystal violet from the membranes. Next, 200-μL aliquots of crystal violet-ethanol suspensions were taken in triplicate from each well and transferred to a 96-well plate to measure the OD_570_ with a microplate reader (Bio-Rad model 550, Bio-Rad, USA). Negative controls consisting of EG or EGM scaffolds in the absence of the fecal microbiota were also included. The OD_570_ values from wells containing EG or EGM and the fecal microbiota were adjusted by subtracting the values obtained from negative controls.

### DAPI imaging by confocal laser microscopy.

Both EG and EGM incubated with fecal microbiota at different time points (i.e., 24, 48, and 72 h) were stained with DAPI to obtain a three-dimensional visualization of the microbial biofilms formed on the scaffolds. Images were acquired using a Nikon A1 Confocal Microscope (Nikon, Japan) equipped with a ×10 objective lens. For DAPI staining, supernatants were removed, and the wells were washed three times with 1 mL sterile PBS to ensure the removal of non-adhered microorganisms. Adhered microbial communities were fixed by adding 1 mL of 2% (wt/vol) paraformaldehyde (PFA, Merck KGaA) and incubated for 16 h at 4°C protected by light. After the removal of PFA, wells were washed three times with 1 mL PBS. Fixed samples were stained by adding 1 mL DAPI (1 μg/mL in PBS) to each well in a dark room. After a 4-h incubation protected by light, DAPI was removed, and the wells were covered with 1 mL PBS. Samples were immediately visualized by confocal microscopy.

### DNA extraction from EG and EGM.

Supernatants were removed from each well, and microbial DNA was extracted from the EG and EGM scaffolds using the phenol-chloroform method. Each membrane was separately transferred to a sterile Falcon tube, resuspended in 5 mL of TES buffer (EDTA 5 mM, NaCl 50 mM, Tris HCl 30 mM [pH 8]), and centrifuged at 4,500 rpm for 10 min. Supernatants were removed and pellets incubated for 1 h at 37°C in 5 mL TES buffer, 1 mL lysozyme (10 mg/mL), and 250 μL RNase (10 mg/mL). Next, 1.05 mL Triton X-100 (8% vol/vol) and 10 μL proteinase K (10 mg/mL) was added before a further 1 h-incubation at 37°C. After incubation, 1.5 mL NaCl 5M and 1.25 mL CTAB/NaCl (10% vol/vol CTAB, 0.7 M NaCl) was added. Next, 500-μL aliquots were taken from each Falcon tube, transferred to sterile Eppendorf tubes, and incubated for 10 min at 65°C. Each tube was then supplemented with 500 μL of a 24:1 chloroform-isoamyl alcohol solution and centrifuged at 14,000 rpm for 10 min. Supernatants were then transferred to clean tubes and 500 μL of a 25:24:1 phenol-chloroform-isoamyl alcohol solution was added. After centrifugation at 14,000 rpm for 10 min, supernatants were transferred to clean tubes, 500 μL of the 24:1 chloroform-isoamyl alcohol solution was newly added, and tubes were centrifuged at 14,000 rpm for 10 min. Supernatants were then transferred to clean tubes, and a volume of isopropanol corresponding to 60% of the supernatant volume was added to facilitate nucleic acid precipitation. Samples were centrifuged at 14,000 rpm for 10 min and the supernatants were removed. In the end, pellets were washed with 1 mL of 70% (vol/vol) ethanol by centrifugation at 14,000 rpm at 4°C for 10 min and resuspended in 50 μL sterile water. Extracted DNAs were subsequently quantified using a NanoDrop Lite spectrophotometer (Thermo Fisher Scientific) and normalized to a standard concentration of 5 ng DNA/μL.

### Real-time qPCR.

Absolute abundances of total bacterial load and each of the main phyla (i.e., *Firmicutes*, *Bacteroidetes*, *Actinobacteria*, and *Proteobacteria*) and genera (i.e., *Akkermansia*, *Bacillus*, *Bacteroides*, *Bifidobacterium*, *Clostridium*, Escherichia*-Shigella*, *Faecalibacterium*, *Lactobacillus*, *Prevotella*, and *Ruminococcus*) were assessed in extracted DNA by 16S rRNA gene-targeting qPCRs. Different primer pairs targeting phylum- or genus-specific 16S rRNA gene regions were selected (listed in the supplemental material; Tables 2 and 3). To evaluate total bacterial abundance, a primer pair targeting a sequence of the 16S rRNA gene conserved in all bacteria was used. qPCRs were performed using a CFX96 Real-Time System (Bio-Rad) and CFX Maestro Software (version 2.3, Bio-Rad). All reactions were carried out in duplicate in a 96-well plate with a final volume of 20 μL per well, including 8 μL sterile water, 0.5 μL of each primer (10 μM), 10 μL of Luna Universal qPCR Master Mix (New England BioLabs, USA), and 1 μL of 5 ng DNA/μL template DNA. The amplification protocol was as follows: an initial denaturation step at 95°C for 1 min, followed by 45 cycles composed of a denaturation step at 95°C for 15 s, an annealing step at each primer set-specific temperature (Tables 2 and 3) for 30 s, and an extension step at 72°C for 10 s. Absolute quantifications were performed by comparison with calibration curves generated using external standards with known concentrations subjected to 10-fold serial dilutions ranging from 10^2^ to 10^10^. For each standard curve, the R^2^ coefficient was higher than 0.98.

**TABLE 2:** Primer pairs used for the quantification of total bacterial load and microbial phyla

Bacterial group	Primer name and sequence (5′–3′)	Amplicon length (bp)	Annealing temp (°C)	Reference
Total bacteria	F: ACTCCTACGGGAGGCAGCAG	200	60	19
	R: ATTACCGCGGCTGCTGG			
*Firmicutes*	F: ATGTGGTTTAATTCGAAGCA	126	62	19
	R: AGCTGACGACAACCATGCAC			
*Bacteroidetes*	F: CATGTGGTTTAATTCGATGAT	126	62	19
	R: AGCTGACGACAACCATGCAG			
*Actinobacteria*	F: CGCGGCCTATCAGCTTGTTG	600	67	45
	R: CCGTACTCCCCAGGCGGGG			
*Proteobacteria*	F: CATGACGTTACCCGCAGAAGAAG	195	63	19
	R: CTCTACGAGACTCAAGCTTGC			

**TABLE 3:** Primer pairs used for the quantification of microbial genera

Bacterial group	Primer name and sequence (5′–3′)	Amplicon length (bp)	Annealing temp (°C)	Source or reference
*Akkermansia*	F: CAGCACGTGAAGGTGGGGAC	329	50	46
	R: CCTTGCGGTTGGCTTCAGAT			
*Bacillus*	F: GCAACGAGCGCAACCCTTGA	92	68	47
	R: TCATCCCCACCTTCCTCCGGT			
*Bacteroides*	F: GAGAGGAAGGTCCCCCAC	106	60	48
	R: CGCTACTTGGCTGGTTCAG			
*Bifidobacterium*	F: CTCCTGGAAACGGGTGG	550	55	49
	R: GGTGTTCTTCCCGATATCTACA			
*Clostridium*	F: AAAGGAAGATTAATACCGCATAA	722	57	50
	R: ATCTTGCGACCGTACTCCCC			
Escherichia *-Shigella*	F: GAGTAAAGTTAATACCTTTGCTC	203	52	This study
	R: ACTCAAGCTTGCCAGTATCAG			
*Faecalibacterium*	F: GGAGGAAGAAGGTCTTCGG	248	50	48
	R: AATTCCGCCTACCTCTGCACT			
*Lactobacillus*	F: GAGGCAGCAGTAGGGAATCTTC	126	65	48
	R: GCCAGTTACTACCTCTATCCTTCTTC			
*Prevotella*	F: GGTTCTGAGAGGAAGGTCCCC	121	60	48
	R: TCCTGCACGCTACTTGGCTG			
*Ruminococcus*	F: GGCGGCYTRCTGGGCTTT	451	63	51
	R: ACCTTCCTCCGTTTTGTCAAC			

### 16S rRNA gene sequencing and metagenomic analyses.

16S rRNA gene sequencing and subsequent data processing were performed by Novogene (Beijing, China). 16S rRNA gene regions V3 to V4 were amplified with the primers 341F (5′-CCTAYGGGRBGCASCAG-3′) and 806R (5′-GGACTACNNGGGTATCTAAT-3′). PCR products were detected with 2% agarose gel electrophoresis and purified with the Qiagen Gel Extraction kit (Qiagen, Germany). Sequencing libraries were generated using the NEBNext Ultra DNA Library Prep kit for Illumina (New England BioLabs). Their quality was evaluated with a Qubit version 2.0 fluorometer (Thermo Fisher Scientific) and the BioAnalyzer 2100 System (Agilent Technologies, USA). Libraries were sequenced on the HiSeq Illumina platform and 250-bp reads were generated. Raw data were filtered using QIIME (version 1.7.0). OTUs were clustered with a ≥97% similarity cutoff using UPARSE (version 7.0.1001). Representative sequences of each OTU were then analyzed using the GreenGene Database, based on the RDP classifier algorithm (version 2.2). Phylogenetic relations between OTUs were assessed with MUSCLE (version 3.8.31). Alpha and beta-diversity analyses were performed using QIIME and R (version 2.15.3).

### Statistical analyses.

For each experiment, five biological replicates were performed. Data are expressed as mean ± standard deviation. All statistical analyses were performed using GraphPad Prism (version 9.3.1, GraphPad Software Inc., USA). Statistical significance was set at *P* < 0.05. Student’s *t* tests for unpaired data were performed to compare measurements obtained from EG and EGM scaffolds at each time of incubation for both crystal violet assays and real-time qPCRs.

### Data availability.

The data sets generated in this study are available at https://www.ncbi.nlm.nih.gov/sra/PRJNA973939.
